# The Current Crisis in Emergency Care and the Impact on Disaster Preparedness

**DOI:** 10.1186/1471-227X-8-7

**Published:** 2008-05-01

**Authors:** Robert A Cherry, Marcia Trainer

**Affiliations:** 1 Department of Surgery, Section of Trauma and Surgical Critical Care, Penn State College of Medicine, Hershey, Pennsylvania, USA; 2 Graduate Program in Public Health Preparedness, Penn State College of Medicine, Hershey, Pennsylvania, USA

## Abstract

**Background:**

The Homeland Security Act (HSA) of 2002 provided for the designation of a critical infrastructure protection program. This ultimately led to the designation of emergency services as a targeted critical infrastructure. In the context of an evolving crisis in hospital-based emergency care, the extent to which federal funding has addressed disaster preparedness will be examined.

**Discussion:**

After 9/11, federal plans, procedures and benchmarks were mandated to assure a unified, comprehensive disaster response, ranging from local to federal activation of resources. Nevertheless, insufficient federal funding has contributed to a long-standing counter-trend which has eroded emergency medical care. The causes are complex and multifactorial, but they have converged to present a severely overburdened system that regularly exceeds emergency capacity and capabilities. This constant acute overcrowding, felt in communities all across the country, indicates a nation at risk. Federal funding has not sufficiently prioritized the improvements necessary for an emergency care infrastructure that is critical for an all hazards response to disaster and terrorist emergencies.

**Summary:**

Currently, the nation is unable to meet presidential preparedness mandates for emergency and disaster care. Federal funding strategies must therefore be re-prioritized and targeted in a way that reasonably and consistently follows need.

## Background

Many assumptions regarding the nation's need for disaster preparedness were reassessed after 9/11. Among them was a fuller appreciation of the fact that preparedness had to include public health and hospital personnel in its first responder definition. This significance was statutorily recognized with the Homeland Security Act (HSA) of 2002[1] which provided for the designation of a critical infrastructure protection program. This subsequently led to the Homeland Security Presidential Directive-7 (HSPD-7)[2] in 2003 which targeted emergency services as a critical infrastructure. The disaster of Hurricane Katrina in 2006 reaffirmed the need for this declaration. Among the lessons learned, it was determined that better planning and integration of emergency services at all levels of government – national, state, regional, and local – was essential for public health preparedness. Certain core questions must therefore be asked: What are our priorities? What assessments have been made to evaluate these priorities? Finally, given finite resources, what tradeoffs have been made in emergency care [3]? In the context of an evolving crisis in hospital-based emergency care, the potential solutions to these questions have become even more challenging, especially given the limitations in federal resources.

## Discussion

A basic priority for our country is a unified emergency response to disasters. The National Response Plan (NRP) [4] and its successor, the National Response Framework (NRF) [5] are designed to coordinate the inherent challenges of America's federalist system, and attempt to balance power and responsibilities of governance between national and various state and local interests [6]. The NRP and the NRF are intended to be a companion to the federal organizational management template, the National Incident Management System (NIMS) [7], and to the Planning Scenarios [8], which are templates for action during certain catastrophic events (Catastrophic Incident Supplement [CIS]), so that the nation's collective responders can be unified in coordinating disaster responses, locally first and then by scaling up [4,7-9].

Another earlier-recognized priority is the nation's need for an organized emergency trauma response. Toward that goal, the Trauma Care Systems Planning and Development Act of 1986 was passed to build the trauma system infrastructure. This was a specific response to a Government Accounting Office (GAO) Report in 1986 revealing that survival rates of rural and urban communities could be dramatically improved through the systematic coordination of a trauma response plan [10]. Trauma is a leading killer and is the primary cause of death for Americans under the age of 44 [11]. Trained trauma centers have contributed to lowering the rate of death from major injury by up to 70%[12]. These facts represent the trauma system's impact on emergency care. Trauma systems also enhance a community's emergency medical response and surge capacity during times of disaster.

Whether of natural or human origin, disasters often involve casualties suffering from extensive trauma, or those injuries caused by physical force [13]. Injuries resulting from chemical, biological, or radiological disasters due to human origin are rare. For example, within the United States alone, there were 31,110 illegal bombings that occurred between 1983 and 2002. Yet, during that same span period, the US experienced only two biological attacks, and no nuclear or chemical attacks [14]. Furthermore, commonly occurring natural disasters, such as hurricanes, earthquakes, and tornadoes, also tend to cause traumatic injuries and typically do not have a biological, chemical, or radiological component. Therefore, the national focus on weapons of mass destruction since 9/11, whether in the media or in the level of federal funding, appears to be misdirected, especially considering the types of threats historically experienced [14].

States with a fully functioning trauma system are in a better position to respond because essential readiness elements are already in place. The day-to-day operations of trauma centers are already structured to meet the strenuous demand of trauma victims by virtue of their expanded capabilities. Trauma centers have the personnel and resources necessary to evaluate and treat large numbers of injured patients on a daily basis. These facilities have highly trained and specialized staff that are capable of rapid decision-making, and have the prerequisite relationships and liaisons with emergency medical services, community hospitals, public health officials, the military and local government[12]. Transportation assets, memorandums of understanding, and educational programs are also essential elements already in place. Thus, during a disaster, trauma centers are better able to scale up their daily operations irrespective of the disaster mechanism [12], and should be the foundation of any disaster medical response [14].

Initially, after the 1986 GAO Report, Congress responded with grant funding for trauma systems. This funding has lapsed several times since then [15]. Despite these gaps in funding after the 1986 trauma act, the federal government continued to recognize the need for a trauma readiness system. In 2001, even before 9/11, the Health Resources and Services Administration (HRSA) had developed a standard survey for states and communities to self-assess their trauma readiness [16]. This standardized questionnaire was used in a 2002 study by Mann and colleagues to assess all fifty states on their trauma needs. These results, published in 2004, provided an essential baseline for evaluating national disaster preparedness. The authors acknowledged that the urgency created by the 9/11 terrorist attacks improved readiness in many states.

The federal need for readiness measures continued. On December 17, 2003, Presidential Directive 8 "National Preparedness" (HSPD-8) [17] was issued and outlined, in areas identified in a Target Capabilities List (TCL), those priority actions that must be taken to improve the nation's preparedness [8]. In the TCL, HRSA specifically addressed surge capacity as a focus and listed it in the majority of its priorities and critical benchmarks [18]. This presidential directive followed a March 2003 General Accounting Office report to the Senate Committee on Finance describing a nationwide trend of overcrowding in emergency departments (EDs). The report discussed long patient waits and increased pain and suffering [19]. Interestingly, HSPD-8 did not address this issue. Later in 2006, however, HRSA revised its survey, and supplied benchmarks, indicators and scoring systems [20]. This report recognized the nation's trauma system capability as a readiness priority. The problem is that sufficient funding must also follow.

**Figure 1:**
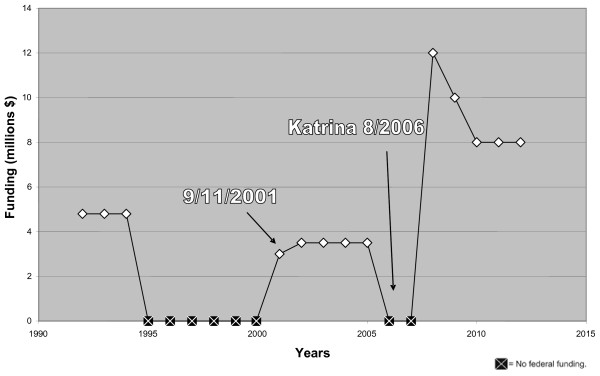
History of federal funding for trauma EMS [24, 25].

Trauma centers are expensive to maintain. They must operate 24 hours a day, 7 days a week, and provide highly trained trauma teams for critical and highly complex injuries. These trauma response teams are comprised of specially trained nurses; up to 16 physicians who specialize in a wide range of fields including trauma surgery, emergency medicine, neurosurgery, orthopedic surgery, anesthesiology, critical care medicine, and radiology. These response teams are also composed of nurses, respiratory therapists, radiology technicians, blood bank personnel, and operating roomstaff [12]. The expense and complexity of this level of readiness is effective and is appreciated by all public agencies in all states and at all levels [21]. A trauma team response is also expected by the clear majority of America's public. A recent study showed that 61% of the public was confident that they would receive the best trauma care, and would feel extremely concerned if that were not the case [22]. Federal funding for HRSA's trauma-EMS program was funded post-9/11 at $3.5 million for 2002, 2003, 2004, and 2005, but cut altogether for fiscal years (FY) 2006 and 2007 [23,24] (See Figure 1).

Recent literature and formal readiness evaluations at all levels – local, state, and federal – show that the nation has suffered a serious decline in surge capacity, despite the presidential declaration of preparedness criticality. According to a recent extensive assessment by the Institute of Medicine, the nation's hospital-based emergency care is now "at the breaking point"[25]. Some statistics convey a functioning system. A national inventory of the trauma system done in 2003 indicated that trauma centers numbered 1154 and representing all levels of trauma categories (Levels I through V) [26]. A 2005 study indicated that an estimated 69.2% of all U.S. residents could access a Level I trauma center within 45 – 60 minutes, and an estimated 84.1% were within 45 – 60 minutes of a Level II trauma center [27].

**Figure 2:**
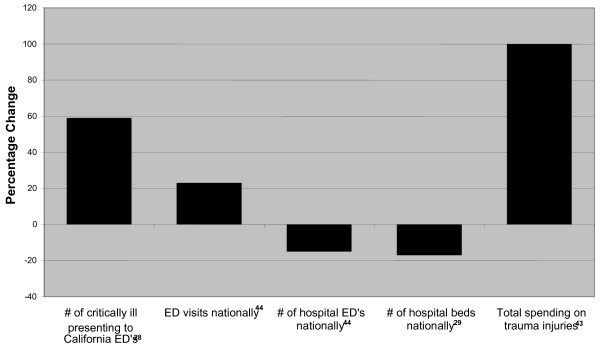
Trends relating to ED/trauma treatment during period of federal elimination 1995–2000.

Unfortunately, these figures do not tell the full story of what happens inside the trauma center. A different picture of hospital emergency departments show a characteristic crisis of overcrowding, boarding, diversions, ambulance bypasses up to 50% of the time, medical care delivered in hallways, makeshift examination rooms, and increased risk of medical error [28]. Such conditions reflect other figures occurring between 1993 and 2003: a 17% decline of hospital beds; a 26% increase in ED visits per year; and a decline of 9% in hospitals with EDs [29] (see Figure 2). These conditions were the topic of a 2005 anecdotal state-by-state survey of expert panels which reported that all states highly valued their trauma systems, but were concerned that a failure of funding was severely impacting the viability of those systems and of the states' healthcare response overall [21].

This nationally recognized crisis is the result of a history of trade-offs. The underlying causes are multiple, complicated, and reflect decades of public policy and corporate business decisions, including health care funding sources, policies regarding insurance, shortages of physician specialists and trained nurses, and issues of liability and who bears the costs[12,25,28,39]. Another significant issue is the lack of training between governments, agencies, and responders [30,31]. The issue of funding has a deep public and private organizational history that has been generated by business decisions, and imbedded in policy assumptions and choices at many levels of public health governance. Managed-care business decisions begun in the 1980's, and spearheaded by cost containment policies, had a direct effect in cutting inpatient beds to assure and sustain a high census [32]. Policies designed to reduce patient treatment time, increase patient throughput, and maximize profit margins, led to a reduction in hospital beds and drastically impacted emergency surge capacity [28]. The average availability of beds for hospitals nationally is 3 to 6% [32]. Restructuring through hospital mergers and closures has also contributed to the reduction in the number of EDs.

Yet another cost-cutting factor affecting disaster preparedness has been the restructuring of hospitals based on the business-systems principle of just-in-time (JIT) inventory management [33]. This form of inventory control restricts a hospital's ability to store equipment and supplies in substantial numbers. Surge capacity is dependent on the "3S's" of staff, stuff, and structure [34]. When the "stuff" required for day-to-day hospital operations are ordered on a JIT basis, unexpected surge requirements may not be met, because on-site storage of supplies and equipment becomes depleted. EDs must then rely on other resources, whether from suppliers on short-notice, other hospitals, or even government stockpiles as a last resort [33]. JIT inventory management potentially compromises the resilience in a system, challenging not only access to essential "stuff" required for surge, but also the staff, who must work under additional stress in an already stressed work environment [35].

The effects of these policy actions place an additional burden on the remaining EDs [28]. Health care insurance programs and policies have impacted the crisis as well and have left many Americans unable to afford private health care coverage. Many of these people are reliant on Medicaid to pay for health care or are simply uninsured [12]. EDs and trauma centers have become safety nets for those who cannot receive treatment elsewhere. This is because EDs are mandated by the 1986 Emergency Medical Treatment and Active Labor Act (EMTALA) to treat and stabilize patients irrespective of their ability to pay [25].

A fairer distribution of insurance stakeholder responsibility would alleviate the situation. The existing fragmentation and cost-cutting policies of healthcare expense-sharing between private insurance companies, managed care plans, federal Medicare, state Medicaid, state workers' compensation, auto insurance companies, and various sources of government funding are ripe for reform [12]. Caught in the middle are EDs and trauma centers which are left accruing the debt for mandatory treatment to the underinsured and uninsured [29]. The emphasis by the federal government to provide funding for a possible response to a biochemical attack, rather than on the trauma centers' recognized role in treating serious injuries from such an attack, is also a serious concern [14].

Specialists who take emergency call are also bearing financial burdens by treating patients who are unable to pay for their care. Uncompensated care is a primary reason that emergency specialists are withdrawing from or declining to elect trauma center employment. Some ED physicians report that 55% of their treatment time is uncompensated [36]. This negative financial impact is due in part to the extraordinary costs of malpractice liability insurance for specialists who provide on-call emergency services to inherently risky, high acuity patients [37]. These EMTALA-mandated safety net hospitals, and their trauma surgeons, emergency medicine physician, and other medical and surgical specialists, become subject to a "dumping ground" syndrome for those hospitals unwilling to incur liability exposure from such high-risk patients who require care [25]. There is a critical national shortage of trauma surgeons and other specialists who are willing to take emergency call because it is fraught with disincentives [37]. Decreasing reimbursement, increasing malpractice costs and medical liability, and an unfavorable lifestyle are among the reasons cited [37,39].

The GAO has published several reports on the continued crisis of recruiting and retaining sufficient nurses, especially as the current population of nurses continue to ages and retire, and the Baby Boomers enter and swell the populations of the elderly [38]. The issue is exacerbated with regard to nurses trained in emergency care because of the stress and burnout rate associated with the practice [39]. Again, cost-cutting policies have contributed to the current shortage of nurses and have led to heavy workloads, difficult staff-to-patient ratios, inadequate wages, and the increasing use of overtime [38]. In addition, the lack of nursing educators have dimmed the prospects of alleviating the staffing shortage in the short-term. Cited are the high costs of obtaining advanced education, and the difficulty that colleges and universities have in funding cost-of-living adjustments for their faculty, including maintaining health care benefits for all of their employees [40]. Federal loan incentives for prospective nurses and nursing educators could alter the trend, both in the short- and long-term.

At all levels of our local, regional and national disaster response plan, there are still problems with a lack of clarity and vision for the future, as well as interagency coordination of preparedness efforts. Jurisdictional boundaries, roles, responsibilities, protocol and communication are all recurring issues in real and simulated disaster responses. Practice is important- and perhaps even more important than the plan [41]. However, practice is expensive, and prohibitively so for the emergency departments and trauma centers because of the lack of funds, available staffing, and the time required to plan and implement training [25]. Without planning and practice, however, the shortcomings of a region's disaster readiness are not realized. Los Angeles recently learned this through a self-selected survey. Even without the benefit of a rehearsed enactment, the surveyed response showed that an effective response would be confined by a "failure to fully integrate interagency training and planning, a failure to develop mutual aid agreements, and a severely limited surge capacity" [30]. The federal government also recognizes that lessons learned from prior practice are essential for future preparedness. This is why it sponsors an annual terrorism exercise required by Congress, called TOPOFF, for the top officials in the Departments of Defense and Homeland Security. Yet, even as the $25 million 2007 TOPOFF exercise was preparing for action in October 2007, Congress was asking for, and had not yet obtained, the 2005 TOPOFF after action report [31]. Preparedness cannot benefit if all levels of participation cannot build upon prior lessons learned.

On the other hand, the comprehensive and compelling 2006 Institute of Medicine report on the crisis in hospital-based emergency care did have an impact. Congress recently renewed funding for the Trauma-EMS program through FY 2012, beginning with $12 million for FY 2008, $10 million for FY 2009, and $8 million for FY 2010 – 2012 [24]. However, these figures are woefully inadequate and unrealistic to remedy a national system of emergency care that has experienced years of budget-tightening policies that have contributed to the current state of crisis. This funding also pales in comparison to the FY 2007 budget for the Department of Health and Human Services which totaled $698 billion [42]. Moreover, the allocated funds diminish through 2012 and will be distributed as competitive grants [24]. This strategy will tend to favor those states with well developed trauma systems and effectively penalize those with an underdeveloped trauma system [8].

## Summary

In our current healthcare system, emergency departments have not only become the primary access point for universal health care, but represent the ultimate safety net for emergency care [37]. Chronic problems involving ED overcrowding and staffing shortages have become unsustainable in the long run and are compromising disaster preparedness efforts. By all measures, the current deficiencies in emergency care are a national crisis [43]. At the same time, the U.S. healthcare delivery system is designated as a critical infrastructure by Presidential directive [8]. Emergency care is the essential bedrock for an all-hazards disaster response [32]. Nevertheless, federal funding for this targeted resource has been suboptimal, both in consistency and in sufficiency. Currently, the nation is unable to meet presidential preparedness mandates for infrastructure readiness, as well as the public's expectations for emergency care. Federal funding strategies must therefore be re-prioritized and targeted in a way that reasonably and consistently follows need.

## Competing interests

The authors declare that they have no competing interests.

## Authors' contributions

RAC was involved in the conception and design of the manuscript, making revisions critical for important intellectual content, and providing final approval of the version to be published. MT has made substantial contributions to the drafting of the manuscript, and the acquisition and analysis of the data presented.

## Pre-publication history

The pre-publication history for this paper can be accessed here:


